# Tackling Few-Shot Challenges in Automatic Modulation Recognition: A Multi-Level Comparative Relation Network Combining Class Reconstruction Strategy

**DOI:** 10.3390/s24134421

**Published:** 2024-07-08

**Authors:** Zhao Ma, Shengliang Fang, Youchen Fan, Shunhu Hou, Zhaojing Xu

**Affiliations:** 1 Graduate School, Space Engineering University, Beijing 101416, China; nuaamazhao@163.com (Z.M.); hshhsc2022@163.com (S.H.); zhaojing3973@163.com (Z.X.); 2 School of Aerospace Information, Space Engineering University, Beijing 101400, China; love193777@sina.com

**Keywords:** few-shot learning, automatic modulation recognition, relation network, deep learning

## Abstract

Automatic Modulation Recognition (AMR) is a key technology in the field of cognitive communication, playing a core role in many applications, especially in wireless security issues. Currently, deep learning (DL)-based AMR technology has achieved many research results, greatly promoting the development of AMR technology. However, the few-shot dilemma faced by DL-based AMR methods greatly limits their application in practical scenarios. Therefore, this paper endeavored to address the challenge of AMR with limited data and proposed a novel meta-learning method, the Multi-Level Comparison Relation Network with Class Reconstruction (MCRN-CR). Firstly, the method designs a structure of a multi-level comparison relation network, which involves embedding functions to output their feature maps hierarchically, comprehensively calculating the relation scores between query samples and support samples to determine the modulation category. Secondly, the embedding function integrates a reconstruction module, leveraging an autoencoder for support sample reconstruction, wherein the encoder serves dual purposes as the embedding mechanism. The training regimen incorporates a meta-learning paradigm, harmoniously combining classification and reconstruction losses to refine the model’s performance. The experimental results on the RadioML2018 dataset show that our designed method can greatly alleviate the small sample problem in AMR and is superior to existing methods.

## 1. Introduction

AMR is an important step between signal detection and demodulation [1]. As a key technology in the field of cognitive communication, AMR is a prerequisite for achieving efficient spectrum sensing and understanding, and it is widely used in wireless monitoring, signal interception and interference problems [2].

For a long time in the past, maximum likelihood theory and expert feature extraction were the main methods for solving AMR problems. Based on the maximum likelihood theory method, it is necessary to first obtain the statistical characteristics of the modulated signal, then construct a decision criterion and finally construct a maximum likelihood classifier. In theory, this type of method can bring high recognition accuracy, but its high computational complexity and strong dependence on prior knowledge limit its application in practical problems. The method based on expert features relies on expert knowledge in the field of signal processing, designing feature-extraction models that can distinguish different modulation signals manually, and then designing classifiers for recognition. The recognition accuracy of this type of method depends on the extracted statistical features and is limited by the weak learning ability of traditional classifiers [3].

In recent years, deep learning technology has shown excellent feature-extraction and data-analysis capabilities in fields such as image processing and natural language processing, and it has been widely applied in wireless communication [4]. At the same time, it also provides a data-driven research approach for AMR. The AMR method based on deep learning utilizes a deep neural network model to learn data distribution from a large number of historical signal samples, extract more robust and effective signal features and use them to distinguish different modulation categories. Recent research has shown that signal modulation-recognition methods based on deep learning have higher recognition performance compared to traditional methods [5]. 

However, most DL-based AMR studies assume sufficient historical signal samples for training deep neural network models and assume that the sample categories are the same when training offline and deploying applications in practice. In practical scenarios, fulfilling this assumption is inherently difficult. Firstly, this is due to the dynamically complex electromagnetic environment, which constantly faces interference from a multitude of sources: various wireless communication devices, different electronic instruments and the ever-changing influences of natural phenomena, including weather fluctuations and terrain characteristics. This continuous change leads to rapid fluctuations in signal characteristics, making it difficult to obtain widely applicable and high-quality signal samples as the samples collected at one time may not represent the situation at other times. Secondly, the collection of high-quality signal samples often requires high-performance measurement equipment and professional data-collection techniques, which brings significant economic and operational burdens. The signal samples also require precise labels for model training, but obtaining accurate labels is also a challenging task. This not only requires a high level of professional knowledge but may also involve a lot of manual work, especially when facing diverse signal types generated by complex electromagnetic environments [6]. Therefore, in practical scenarios, AMR often faces the few-shot challenge, which greatly limits the implementation and widespread application of deep learning-based signal modulation-recognition methods in practical scenarios.

In the field of image recognition, the few-shot problem has also received sufficient attention, and methods such as data augmentation and meta-learning have been proposed to solve the few-shot dilemma. Among them, meta-learning is considered the most promising method for solving small sample problems [7]. Its idea is to continuously construct small sample training tasks on the source domain dataset so that the model can distinguish similarities and differences between different categories. Even when facing new small sample tasks in the target domain, it can still have good recognition performance. Metrics-based meta-learning methods, such as prototype networks (PNs) [8] and relation networks (RNs) [9], have been widely studied and applied due to their simple structure, ease of training and good performance.

Therefore, this paper proposes a few-shot AMR method based on a multi-level comparison class reconstruction relation network (MCRN-CR) based on the meta-learning paradigm, aiming to solve the few-shot problem faced in AMR.

The main contributions of this article are as follows:

(1) A meta-learning paradigm is proposed for the FS-AMR problem. By utilizing existing source domain datasets and constructing meta-task training methods, the model can distinguish differences between different categories and can be well generalized to target domain classification tasks. Namely, it can attain satisfactory classification performance on the target domain with either no or minimal training samples from the target domain.
(2) A multi-level embedding comparison relationship network has been proposed. Based on the relation network architecture in meta-learning, we propose an improved version, which involves multi-level measurement of the deep and shallow features output by the embedding function, synthesizing the relationship score of the test sample relative to the support category and using this to determine the category of the test sample. Compared to general relational networks, the comprehensive comparison of deep and shallow features can minimize the confusion of different modulation categories in the metric space as much as possible.
(3) A class reconstruction meta-learning framework was proposed. The class reconstruction network consists of an encoder and a decoder, where the encoder serves as an embedding function and the decoder reconstructs the potential representation of the sample into the original signal. Furthermore, by combining class reconstruction loss and classification loss to jointly train the model, more robust feature representations can be learned, reducing the impact of noise on recognition performance.

The rest of this article is organized as follows. In the second section, relevant works on AMR are introduced, including traditional AMR methods, DL-based AMR methods and FS-AMR methods. In the third section, the relevant theories and methods of this article are introduced. The fourth section provides a detailed introduction to the multi-level comparison relation network proposed in this article, which combines class reconstruction. The fifth section provides detailed experimental results and discussion. Finally, a summary of the entire article was provided. 

## 2. Related Works

This section focuses on the problem of AMR, introducing traditional AMR methods, DL-based methods and existing FS-AMR methods.

### 2.1. Traditional AMR Methods

#### 2.1.1. Maximum Likelihood Theory-Based AMR Method

The modulation-recognition method based on maximum likelihood theory analyzes signal characteristics through probability theory, and its modulation-recognition process is a multi-hypothesis testing problem. This method is based on the likelihood function of electromagnetic signals, using the comparison results of the likelihood ratio and set threshold as the basis for judgment. Common methods include average likelihood ratio test (ALRT) [10], generalized likelihood ratio test (GLRT) [11] and mixed likelihood ratio test (HLRT) [12]. This type of method has the best signal feature performance in ideal channels, but it has high computational complexity and requires complete signal prior knowledge, making it susceptible to environmental interference factors.

#### 2.1.2. Expert Features-Based AMR Method

Feature extraction is a process of data mapping, which involves mapping the original signal data to a specific feature space. The purpose of feature mapping is to generalize the differences between modulation categories, reduce data dimensionality, and improve algorithm efficiency. Common signal features include the following: (1) instantaneous features; (2) statistical characteristics; (3) transform domain features [6].

Instantaneous features describe the instantaneous properties of a signal in the time domain. It mainly considers the instantaneous change characteristics of the signal at a certain moment or period [13]. Common instantaneous characteristics include instantaneous amplitude, instantaneous frequency and phase. Statistical features are obtained through statistical analysis of signals. They describe the probability distribution, average level, dispersion degree and other characteristics of signals, mainly including high-order moments, high-order cumulants and cyclic cumulants [14,15]. The transformation domain feature is acquired through the mathematical transformation of a signal, such as using methods like the short-time Fourier transform or wavelet transform. These methods convert the signal from the time domain to the frequency domain or other transformation domains, revealing valuable information such as the frequency, phase and energy of the signal. The commonly used transform domain features mainly include wavelet transform features, spectral analysis features, time-frequency maps, constellation maps [16], etc.

Feature extraction is fundamental in expert feature-based methods and requires a high level of expertise. The effectiveness of features directly impacts the final classification performance. However, this method is limited by its reliance on a single feature, which constrains its data representation capability and, consequently, caps its performance potential. Additionally, feature extraction and classifier design are conducted separately, resulting in low automation and high time costs.

### 2.2. DL-Based AMR Methods

The deep learning-based AMR method uses deep neural network models for feature extraction and classification. Compared with traditional methods, the deep learning-based method achieves higher recognition accuracy, stronger generalization ability and wider applicability, thus gaining widespread attention. From the perspective of network structure, currently, the types of deep neural networks (DNNs) used for designing modulation recognition include Convolutional Neural Networks (CNNs), Recurrent Neural Networks (RNNs) and their hybrid networks.

#### 2.2.1. CNN-Based AMR Methods

O’SHEA et al. first proposed a modulation-recognition neural network model for directly processing raw In phase and Quadrature (I/Q) signals in 2016. Although this model only uses a simple CNN structure, its recognition performance far exceeds traditional artificial feature methods. At the same time, the author also opened up the RadioML2016.10a and RadioML2016.10b datasets, greatly promoting the development of this field. Furthermore, classic deep learning architectures such as VGG [17] and ResNet [18], which are widely used in the image field, have also been applied to AMR problems. O’SHEA released the RadioML2018.01A dataset in 2018 and compared the classification performance of two types of networks for AMR. The experiment proved that the ResNet structure with skip connections could bring better recognition accuracy under a high signal-to-noise ratio. In addition, the author also compared the recognition performance of the model with different convolutional layers in VGG and different residual block numbers in ResNet and pointed out that a too-deep network architecture not only cannot bring higher recognition performance [19] but also can bring higher complexity and computational complexity, leading to overfitting of the model during training. 

In addition to processing I/Q signals directly, the original I/Q data is typically preprocessed and transformed into other forms of features. Corresponding DNN models are then developed for deep feature extraction and recognition. Constellations [20], spectrograms [21], as well as cyclic spectrograms [22], eye charts [23], amplitude histograms [24], etc., are all used as data preprocessing forms in AMR problems. In contrast, the DL-AMR method with preprocessed form has stronger comprehensibility, but there is additional computational complexity in the process of converting I/Q signals into images. The DL-AMR method, which directly processes I/Q signals, does not require additional preprocessing processes, requires less computation and has a higher degree of automation.

#### 2.2.2. RNN-Based AMR Methods

Note that the modulated signal is a time series, and CNNs typically struggle to capture temporal features well, so RNN-based AMR methods are gradually being proposed. A new AMR method based on RNN is proposed in [25], which uses gated recurrent units (GRUs) to achieve better recognition accuracy than some CNN models. The author of reference [26] converted the I/Q signal into amplitude and phase (A/P) and input it into LSTM, achieving high recognition accuracy. 

#### 2.2.3. Hybrid AMR Method of CNN and RNN

Based on a single CNN or RNN network architecture, it is difficult to simultaneously focus on the spatial or temporal characteristics of wireless signals. Using only one type may not achieve optimal performance. Therefore, some scholars have proposed an AMR model that combines the two network architectures. A Convolutional Long Short Term Deep Neural Network (CLDNN) model is proposed in [26], consisting of one LSTM and three CNN layers. This model has a skip connection before LSTM, bypassing two CNN layers, aiming to provide longer temporal correlation information for the extracted features. Reference [27] proposes another CLDNN2 model without bypass layer connections, which can achieve higher recognition accuracy than CLDNN at the cost of increasing the number of layers and parameters. Inspired by the excellent feature-extraction characteristics of hybrid models and the complementary information present in individual channels, reference [28] proposes a new multi-channel deep learning model (MCLDNN), which utilizes single and combined I/Q symbols of received data to extract features from a spatiotemporal perspective, achieving high recognition performance.

#### 2.2.4. Other Network Architecture-Based AMR Method

In addition to CNN and RNN, popular network structures in deep learning such as Complex Value Neural Networks (CVNN), Graph Neural Networks (GNN) and Transformer have also been used in the study of AMR problems. Compared with traditional real neural networks, CVNN can simultaneously process data containing both real and imaginary parts, providing richer and more diverse feature-expression capabilities than real neural networks [29]. Therefore, CVNN is very suitable for processing periodic signals. Reference [30] proposed a novel AMR model based on CVNN and found that complex-valued operations are more effective in helping neural networks extract correct statistical information than real-valued operations at low signal-to-noise ratios. To reduce the additional computational cost of complex convolution, reference [31] proposes an AMC model based on complex depthwise separable convolution (CDSCNN) to achieve a balance between classification accuracy and model complexity. When the essence of a problem can be represented by a graph structure, a graph neural network (GNN) can be used to solve the problem [32]. Reference [33] first proposed an automatic modulation-recognition model based on Graph Convolutional Neural Network (GCN). Transformer [34] has also been proven to be useful for solving AMR tasks. For example, the Mcformer model in reference [35] achieved good classification performance by extracting global information from time-domain signals through the Transformer.

### 2.3. FS-AMR Methods

Based on different research approaches to solving few-shot problems, we divide them into data-driven few-shot learning methods and model-driven few-shot learning methods.

#### 2.3.1. Data-Driven FS-AMR Methods

The data-driven approach mainly focuses on how to enhance or expand existing small training samples and improve the learning performance of the model under few-shot conditions. When the labeled samples are insufficient, on the one hand, simple data transformations such as cropping and concatenation, and adding noise can be used to expand the training samples. On the other hand, Generative Adversarial Networks (GAN) can be used to generate virtual data. Based on the characteristics of electromagnetic signal data, reference [36] analyzed and applied several simple and effective time-series enhancement methods that conform to the characteristics of electromagnetic signals, including noise disturbance, amplitude and time delay transformation, frequency shift and phase shift. Finally, the effectiveness of the proposed simple data-augmentation method in small sample radiation signal-recognition tasks was verified through experiments. The author of reference [37] used GAN to generate pseudo data similar to the original signal and reversed the original signal and false signal to further increase sample diversity. Finally, the effectiveness of data augmentation based on the GAN method was verified in a CNN network model.

#### 2.3.2. Model-Driven FS-AMR Methods

This type of method focuses on designing and optimizing model structures, learning strategies or optimization algorithms, enabling them to have inherent few-shot learning capabilities and efficiently learn and generalize under limited data. Meta-learning is a commonly used learning framework for such methods, which enables machines to learn to learn and distinguish similarities and differences between things. Metrics-based meta-learning, such as prototype networks [8] and relational networks [9], is a simple and efficient method for solving few-shot problems. Reference [38] proposes a novel small sample learning framework called Attention Relationship Network (AMCRN), which introduces channel and spatial attention to learn more effective feature representations of supporting samples and uses the relationship network as the learning architecture. The experimental results show that even with only one supporting sample, this method can achieve excellent performance in fine-grained signal modulation recognition and is robust to low signal-to-noise ratio conditions. References [39,40] also proposed corresponding small sample modulation-recognition frameworks based on relational networks. Both use one-dimensional convolution and denoising autoencoder as embedding functions for feature extraction. The experimental results show that compared with traditional FSL algorithms, the method based on relational networks can achieve higher classification accuracy in signal modulation recognition. In addition, reference [41] also proposed the AMR-CapsNet method based on capsule networks to solve the problem of few-shot AMR and achieved good classification results.

## 3. Signal Model and Basic Theory

### 3.1. Signal Model

AMR is a prerequisite for signal demodulation. The module for processing modulation classification is usually deployed at the receiving end of the entire communication system, sending the received modulation information to the demodulator, which then demodulates it.

In wireless communication systems, the received signal can be represented as [42]:

(1) X(t)=h(t)×s(t)+n(t),

where *h*(*t*) represents the wireless channel pulse response, *s*(*t*) represents the modulated signal generated by the transmitter, and *n*(*t*) represents the most basic additive Gaussian white noise (AWGN).

After orthogonal sampling by the receiver, the same *I/Q* signal *X*(*n*) is obtained:

(2) $$X(N)=\left[\left(x_{1}^{I},x_{1}^{Q}\right),\left(x_{2}^{I},x_{2}^{Q}\right),\ldots ,\left(x_{N}^{I},x_{N}^{Q}\right)\right],$$

where *N* is the sampling length. Therefore, when *I*/*Q* signals are used as network inputs, their data format can be written as:

(3) $$X_{\mathrm{input}\;}=\left[\begin{array}{cccc} x_{1}^{I} & x_{2}^{I} & \ldots & x_{N}^{I}\\ x_{1}^{Q} & x_{2}^{Q} & \ldots & x_{N}^{Q} \end{array}\right].$$

AMR can be described as determining the modulation method of *s*(*t*) by observing the signal *X*(*t*).

### 3.2. Description of DL-Based AMR Methods

In DL-based AMR tasks, the collected historical signals are usually used as training data, and known modulation methods are used as labels to train deep neural networks and obtain recognition models.

Given the training sample set

(4) $$D=\left\{\left(x_{1},y_{1}\right),\left(x_{2},y_{2}\right),\cdots ,\left(x_{m},y_{m}\right)\right\},\;m=1,2,\cdots M,$$

Among them, *x_m_* is the *m*-th sample data, *y_m_* is the true label of the *m*-th sample, with a value range of label number, $y_{m}\in (0,1,2,\cdots ,L-1)$. The training task is to obtain the optimal parameters of the given model by learning the training sample set under the given optimization objective conditions:

(5) $$\theta =\underset{\left(x_{m},y_{m}\right)\in D}{\arg\min}\sum_{m=1}^{M}\mathrm{loss}\left({\hat{y}}_{m},y_{m}\right)=\underset{\left(x_{m},y_{m}\right)\in D}{\arg\min}\sum_{m=1}^{M}\mathrm{loss}\left({\mathrm{Model}}_{\theta }\left(x_{m}\right),y_{m}\right).$$

Then, by applying the trained model parameters *θ*, substituting them into the Model and testing the test sample set, we can infer the category of the test samples and obtain a measure of the Model’s generalization ability.

However, the actual signal environment is very complex, and various electromagnetic activities interact with each other, making it difficult to obtain sufficient high-quality labeled samples. Therefore, the training samples cannot accurately represent the data distribution, which makes it difficult for deep neural network models to learn appropriate parameters. This results in significant classification model errors and poor generalization ability. This is the few-shot dilemma faced by AMR.

### 3.3. Metrics-Based Meta-Learning

Meta-learning is an important method for solving few-shot problems. Unlike traditional deep learning methods, its purpose is to enable machines to learn to learn and have the ability to distinguish similarities and differences between things, rather than learning a classification model specific to a specific task. We assume that the training set *D*_m-train_ in the source domain and the testing set *D*_m-test_ in the target domain have different categories. The basic idea of meta-learning is to construct meta-tasks on the source domain training set, and through training on different meta-tasks, make the machine have good generalization ability when facing different classification tasks, and thus have good recognition performance on target domain testing tasks.

**Figure 1:**
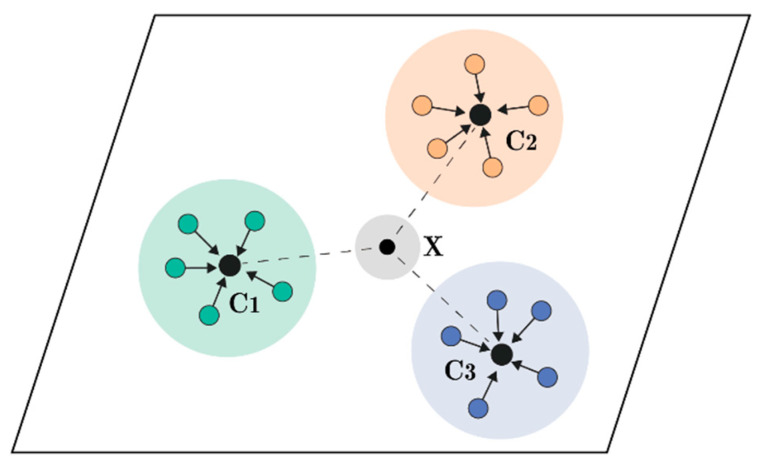
Schematic diagram of measurement space.

Metrics-based meta-learning consists of an embedding function and a metric function defined in the representation space. As shown in Figure 1, the embedding function is used to extract prototype features of different categories, and the measurement function is used to measure the similarity between the test sample and different categories. The higher the similarity, the closer it is to that category. In meta-learning, one training iteration is called an Episodic, which is a *C*-Way *K*-shot classification task. *C*-Way *K*-shot task extraction refers to randomly selecting *C* categories from the training set, with *K* samples taken from each category to form a support set, and then extracting *Q* samples as a query set. The category labels of the support set are known and are used to construct the embedding benchmark of category *C* in the metric space, while the query set is the sample to be predicted.

**Figure 2:**
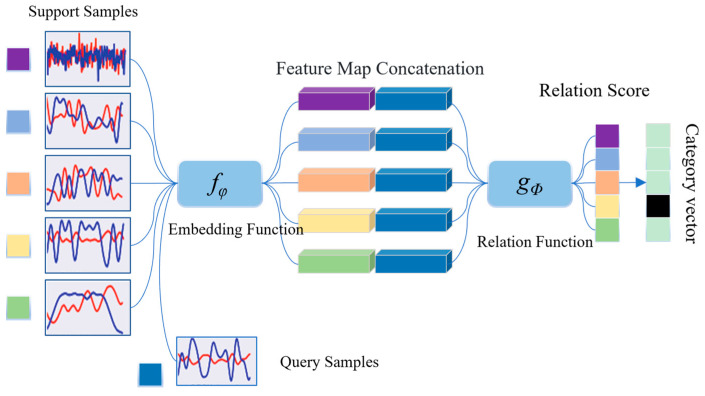
AMR method based on relational networks.

Figure 2 is a schematic diagram of signal modulation recognition using a relational network in a 5-way 1-shot scenario. The embedding function and relation metric function of the relational network are *f_θ_* and *g_φ_*, respectively. Assuming the support sample is *x_i_* and the query sample is *x_j_*, the embedding function calculates the embeddings *f_θ_*(*x_i_*) and *f_θ_*(*x_j_*), and then concatenates the two feature maps:

(6) $$Z\left(f_{\theta }\left(x_{i}\right),f_{\theta }\left(x_{j}\right)\right),$$

and feed the results into the relationship metric function to calculate the relation score, which is the similarity score:

(7) $$r_{i,j}=g_{\varphi }(Z(f_{\theta }(x_{i}),f_{\theta }(x_{j}))).$$

Finally, the category of the query sample will be determined based on the relation score. During the training process, minimize the objective function to seek the optimal solution *θ* related to *φ*, which enables the network to optimize task modeling, and quickly converge when accepting new small sample tasks.

## 4. The Proposed Method

This section will introduce the AMR method based on MCRN-CR in three parts: (1) the framework of MCRN-CR, (2) the details of MCRN-CR and (3) the training steps.

### 4.1. The Framework of MCRN-CR

**Figure 3:**
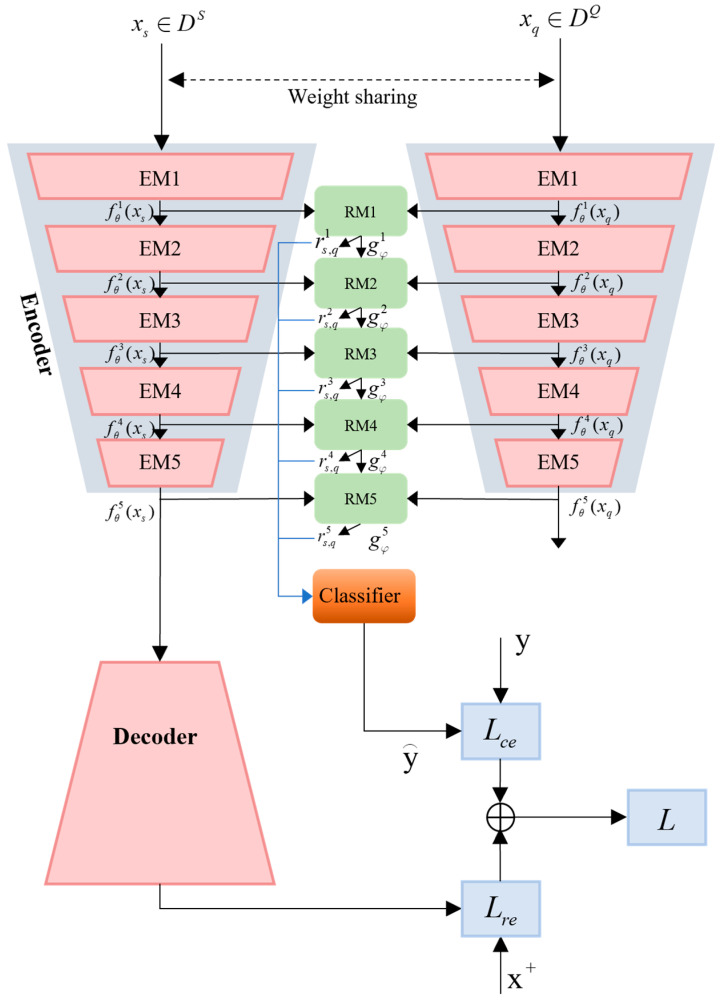
The overall framework diagram of the proposed MCRN-CR.

To solve the few-shot dilemma in AMR, this paper proposes a class reconstruction relation network based on multi-level comparison (MCRN-CR), whose framework structure is shown in Figure 3. MCRN-CR consists of two parts: class reconstruction and multi-level comparison relation network. The class reconstruction part is used to generate low-dimensional latent representations of input samples, which is the embedding of support samples and query samples. The multi-level comparison relation network is an improved version of the relation network, used to achieve recognition tasks under few-shot conditions. It should be noted that Figure 3 is a schematic diagram. Although there are only 4-levels of MCRN-CR in Figure 3, we can construct models at any level. In this article, we construct a 5-level MCRN-CR.

During the training process, the class reconstruction part first generates a low dimensional latent representation **z***_s_* of the support sample **x***_s_*. On the one hand, **z***_s_* participates in the similarity calculation between query samples. On the other hand, it needs to be further fed into the decoder to generate the reconstruction support sample ${\bar{x}}_{s}$. To eliminate the influence of noise, when further calculating the reconstruction loss *L_re_*, a high signal-to-noise ratio sample ${x_{s}}^{+}$ with the same category as the supporting sample is used as the reconstruction target,

(8) $$L_{re}=E\left({\left\|{\bar{x}}_{s}-{x_{s}}^{+}\right\|}_{2}^{2}\right).$$

Then, the classifier in the MCRN part predicts the class labels of the query samples and calculates the cross-entropy classification loss with the real labels:

(9) $$L_{ce}=-E(y\log\hat{y}).$$

The class reconstruction part helps the encoder as an embedding function extract more recognizable sample features, increase the differences between different class embeddings and thus improve signal-recognition performance under few-shot conditions. In MCRN-CR, *L_re_* and *L_ce_* together constitute the model’s loss, with coefficients of *λ_ce_* and *λ_re_*. We believe that classification loss is more important for model training in the classification task of this article; therefore, *λ_ce_* and *λ_re_* is set to 0.8 and 0.2, respectively.

(10) $$L=\lambda_{ce}L_{ce}+\lambda_{re}L_{re}.$$

### 4.2. The Details of MCRN-CR

#### 4.2.1. The Structure of Improved Relational Networks MCRN

We believe that in order to match query samples and support categories accurately, it is necessary to learn nonlinear distances at different feature levels on both deep and shallow abstract features and comprehensively calculate relation scores. Therefore, in response to AMR, this paper proposes a multi-level comparison relation network, as shown in Figure 3. The multi-level comparison relation network includes an embedding module and a relation-measurement module, where the embedding module is composed of the encoder of the class reconstruction part.

Specifically, the support sample and query sample are, respectively, embedded to generate the *v*-th level feature map $z_{i}^{v}=f_{\theta }^{v}(x_{i})$ and $z_{j}^{v}=f_{\theta }^{v}(x_{j})$, then concatenate it into [$z_{i}^{v},z_{j}^{v}$] and send it to the corresponding *v*-level relation module for comparison.

At the *v −* 1 level, the relation module outputs the similarity feature maps of samples *x_i_* and *x_j_*. The *v*-th level relation module takes both the embedding outputs of the *v*-th level support and query samples as inputs and the similarity feature maps of the *v −* 1 level relation module as inputs:

(11) $$g_{\varphi }^{v}=g\left(\left[z_{i}^{v},z_{j}^{v},g_{\varphi }^{v-1}\right]\right).$$

For the first level relationship module, since it does not have the input of the previous one, it is assumed that

(12) $$g_{\varphi }^{1}=g\left(\left[z_{i}^{1},z_{j}^{1}\right]\right).$$

Assuming *q*(.) represents average pooling and fully connected operations, the similarity (relationship) score between the support samples and query samples output by each relationship module in the feature map at level *v* is:

(13) $$r_{i,j}^{v}=q\left(g_{\varphi }^{v}\right).$$

The similarity score between the further query sample *x_j_* and each supporting category *y*_c_ is:

(14) $$r_{c,j}=\sum_{v=1}^{V}w_{j}^{v}r_{c,j}^{v},\;c=1,\;2,\;\ldots ,\;C.$$

Among them, $w_{c,j}^{v}=\alpha^{v}\left(g_{c,j}^{v}\right)$ represents the scalar attention weight of the relation scores at all levels, $\alpha^{v}$ is a fully connected layer, the activation function is sigmoid and the weight parameters of $\alpha^{v}$ are included in the relationship module. 

#### 4.2.2. The Class Refactoring in MCRN-CR

**Figure 4:**
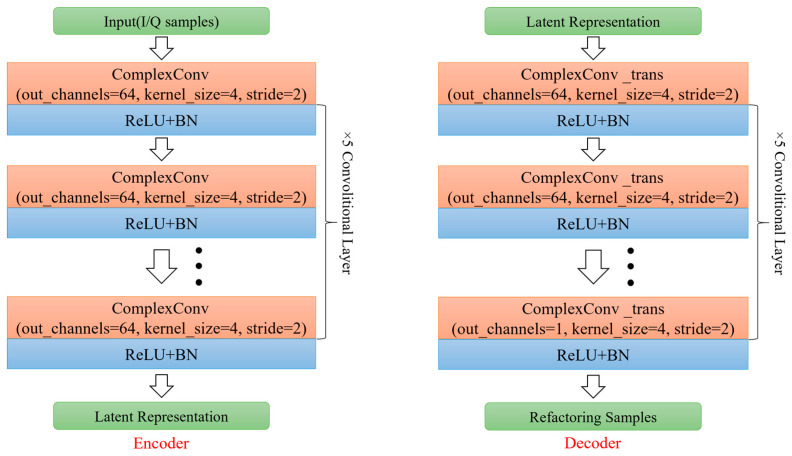
Structure of encoder and decoder.

The class reconstruction part includes two parts: the encoder and the decoder. The encoder generates the potential feature-expression zs of the supporting sample *x_s_*, and the decoder further reconstructs the supporting sample ${\bar{x}}_{s}$. After training, the smaller the reconstruction error, the better zs can represent the sample features. Since complex neural networks can handle data containing both real and imaginary parts, providing richer and more diverse feature-expression capabilities than real neural networks, this paper adopts complex convolution as the basic unit of encoders and decoders. As shown in Figure 4, the structure of the encoder and decoder includes five layers of complex convolution.

#### 4.2.3. Relation Metric Module

The relation metric module calculates the nonlinear distance between query samples and support categories, obtains the relationship score between each sample and different category families and further determines the category of query samples. Table 1 shows the specific structure of the relationship-measurement module.

### 4.3. Training Procedure

During training, C-way K-shot tasks will be extracted from the training set for each iteration, obtaining the support set $D_{S}={\left\{\left(x_{i},y_{i}\right)\right\}}_{i=1}^{m}$ and query set $D_{Q}={\left\{\left(x_{j},y_{j}\right)\right\}}_{j=1}^{n}$ for each training task, where *m* = *K* × *C* and *n* = *Q* × *C*.

Subsequently, according to Section 3.2, calculate the relationship score *r_c_*_,*j*_ between each query sample *x_j_* and each support category *y_c_*, and further determine the predicted label $\hat{y}$ for the query sample. Secondly, the hidden layer feature **z***_s_* of the support sample calculated by the encoder is further fed into the decoder to reconstruct the support sample ${\bar{x}}_{S}$. Then, based on the predicted labels and reconstructed samples, the classification error and reconstruction error are settled separately. Finally, the error is backpropagated, and the encoder and decoder parameters of the class reconstruction part and the relationship module parameters are updated. Algorithm 1 describes the complete training process of MCRN-CR.
**Algorithm 1:** Training process of AMR model based on MCRN-CR.
**Symbol Description:**E: The number of training iterations, the size of Episodic;*Θ*, *θ_de_*, *φ*: Parameters are encoder, decoder and relationship module, respectively;*lr_en_*, *lr_de_*, *lr_rm_*: Learning rates of the encoder, decoder and relation module, respectively;*λ_ce_*, *λ_re_*: a scalar used to balance the overall training loss.
**Training iteration:**1:**For** Episodic = 0 to E − 1:2:  Task Extraction from *D*_m-train_, get Support set *D_S_* and Query set *D_Q_*;3:  x^+^ ← The mean value of the samples corresponding to the category of all supporting samples in the current task at the highest SNR;
  **Forward propagation:**
4:  Calculate the levels of embeddings $z_{i}^{v}$ and $z_{j}^{v}$ for support samples and query samples:
$z_{i}^{v}=f_{\theta }^{v}\left(x_{i}\right),\;\;z_{j}^{v}=f_{\theta }^{v}(x_{j})$;5:  Calculate support category feature embeddings:
   $z_{c}^{v}$ ← The mean embedding of *K* supporting samples in each category;6:  Calculate the relationship score between query samples and support categories:
$r_{c,j}=\sum_{v=1}^{V}w_{j}^{v}r_{c,j}^{v},\;c=1,\;2,\;\ldots ,\;C$;7:  Category labels for predicted query samples:
$\hat{y}=argmax(r_{c,j})$;8:  Calculate the output of the decoder:
$\bar{x}=f(\theta_{de},x_{s})$;9:  Calculate losses:
*L_ce_*$=L_{\mathrm{ce}}(\hat{y},y$), *L_re_*$=L_{\mathrm{re}}(\bar{x},x^{+}$), *L* = *λ_ce_L_re_* + *λ_re_L_re_*;10:  Error backpropagation, updating parameters:
$\begin{array}{l} \theta \leftarrow \mathrm{Adam}\left(\nabla_{\theta },L,lr_{en},\theta \right)\\ \theta_{de}\leftarrow \mathrm{Adam}\left(\nabla_{\theta_{de}},L,lr_{de},\theta_{de}\right)\\ \varphi \leftarrow \mathrm{Adam}\left(\nabla_{\varphi },L,lr_{rm},\theta \right) \end{array}$;11:**End for.**

## 5. Experiment and Discussion

### 5.1. Dataset and Experimental Setup

To verify and evaluate the effectiveness of the proposed method, experiments were conducted on the RML2018.01A modulated signal dataset in this paper. This dataset consists of 24 types of modulation signals, with each signal sample containing two channels of I/Q data in the format of [1024,2]1024 × 2, with a signal-to-noise ratio range of −20 dB~30 dB and an interval of 2 dB. A single category has 4096 samples at each signal-to-noise ratio, totaling 2,555,904 pieces of data.

Firstly, divide the dataset into 14 modulation categories as known source domain training sets and the other 10 as target domain datasets. Due to the large size of the original dataset, it is redundant for the experiment in this article. Therefore, only 1000 samples were taken for each category under a single signal-to-noise ratio. The existing training samples in the target domain are usually scarce in few-shot scenarios. Therefore, to comply with this setting, a subset of the target domain dataset will be divided as a known small sample dataset, with a quantity of 2% of the target domain samples. For each signal-to-noise ratio, 20 samples will be taken for a single modulation method. During each testing process, the support set is extracted from that subset. The remaining part is the test set, from which query samples are extracted during each test. The specific division is shown in Table 2.

The hardware environment for the experiment was as follows: Intel (R) Core (TM) i7-10700k CPU@3.8GHz NVIDIA GeForce GTX3090 GPU. The software environment was as follows: Python 3.8, PyTorch deep learning framework.

### 5.2. Comparison of Recognition Performance with Other Models

To verify the effectiveness and superiority of the proposed method under few-shot conditions, we compared the recognition performance of MCRN-CR with other networks under the 5-way 5-shot setting. The compared models include the most recent FS-AMR methods, AMCRN [40], AMR-CapsNet [42], Prototype Networks (PNs) [8] and Relation Networks (RNs) [9] for few-shot learning methods in the image domain. At the same time, in order to demonstrate that the proposed method can solve the small sample dilemma faced by traditional deep learning methods, a complex neural network-based automatic modulation-recognition model AMR-CVNN is formed by adding a classifier to the structure of the encoder mentioned above, and it is used as a comparison term. To ensure the fairness of the experiment, AMR-CapsNet and AMCRN maintain the same settings as the original text. The laboratory settings for PNs and RNs are consistent with MCRN-CR. AMR-CVNN adopts the idea of transfer learning, first pre-training on the source domain dataset and then fine-tuning the weight parameters on the target domain dataset.

**Figure 5:**
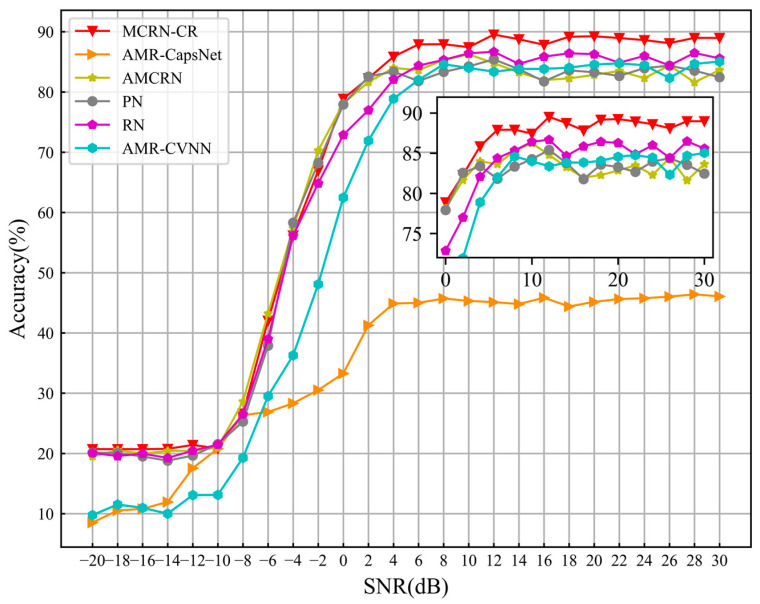
The recognition accuracy of different models at all SNRs.

Figure 5 shows the recognition accuracy of all comparison models on a given dataset under all SNRs. It shows that when the SNR is greater than 0 dB, the proposed method MCRN-CR has the best recognition performance. When the SNR is below 0 dB, the recognition performance of all metric-based meta-learning methods is not significantly different, but the recognition performance of MCRN-CR is much higher than that of AMR CapsNet and AMR CVNN.

Table 3 presents more specific identification performance statistics for each comparative model. The highest recognition accuracy of MCRN-CR reached 89.25%. Under all SNRs, the average recognition accuracy of MCRN-CR reached 65.98%. When SNR > 0 dB, the average recognition accuracy reached 87.41%. Compared to AMR-CVNN, the average recognition accuracy of MCRN-CR has increased by nearly 10% below 0 dB, 5.86% above 0 dB and 8.04% above all signal-to-noise ratios. Therefore, it can be proven that the MCRN-CR proposed in this article can effectively solve the small sample dilemma faced by traditional deep learning methods in AMR problems.

**Figure 6:**
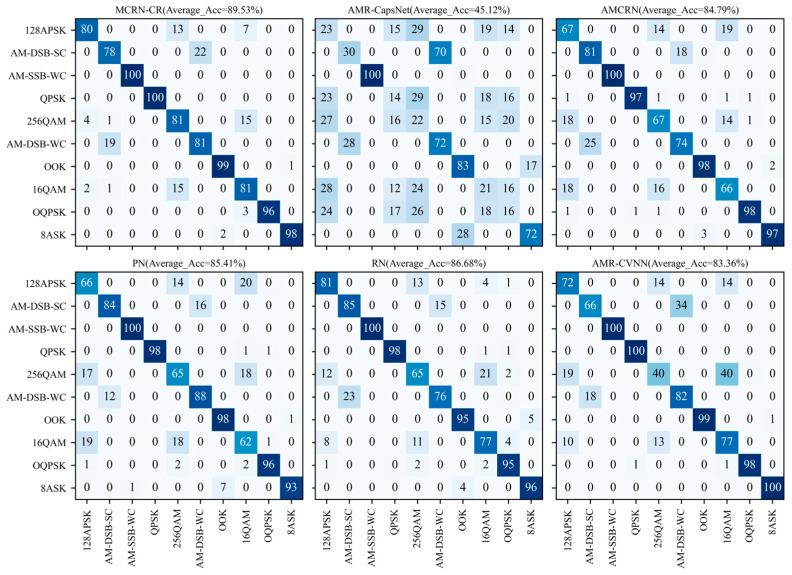
Confusion matrix diagram at the 12 dB SNR.

Figure 6 shows the confusion matrix of all models at the 12 dB SNR. The horizontal axis of the confusion matrix represents the actual modulation category, and the vertical axis represents the modulation category predicted by the model. It can be seen that when distinguishing between high-order digital modulation signals 16QAM and 256QAM, as well as analog signals AM-DSB-SC and AM-DSB-WC, there is still some confusion in MCRN-CR, which is the main reason for the bottleneck in model-recognition performance. The same applies to models other than MCRN-CR. Therefore, we believe that the proposed AMR method based on the meta-learning paradigm can solve the problem of small samples, but for some easily confused signals, it is necessary to design a feature-extraction network with stronger feature-extraction capabilities to extract finer features and achieve the goal of distinguishing confused signals.

### 5.3. Structural Effectiveness Analysis

This section analyzes the impact of multi-level comparison operations and the introduction of class reconstruction modules in the proposed framework. Therefore, four control groups were set up: MCRN-CR, MCRN, RN-CR and RN. Among them, MCRN, RN-CR and RN, respectively, remove the class reconstruction part, the multi-level comparison part and the class reconstruction part and multi-level comparison part.

**Figure 7:**
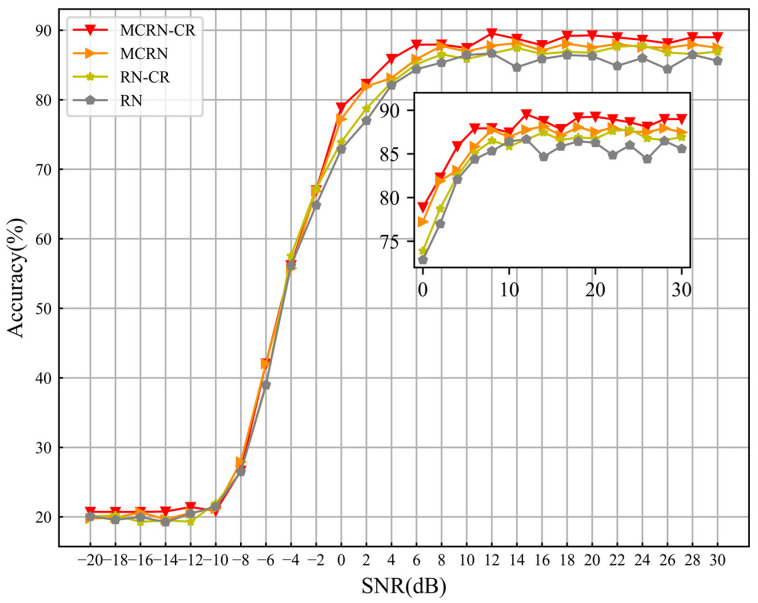
The recognition accuracy of the comparison model at all SNRs.

**Figure 8:**
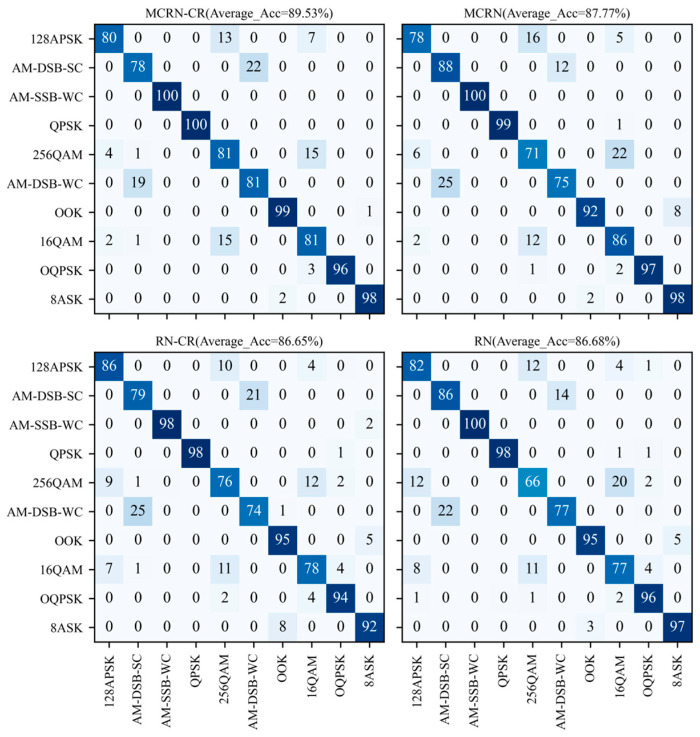
Confusion matrix diagram of the control model at the 12 dB SNR.

Figure 7 shows the recognition performance curves of four control groups at all SNRs, while Table 4 presents further experimental results and statistical data. It can be seen that with the addition of class reconstruction modules and multi-level comparison operations on the basis of the original RN model, the recognition performance has also been correspondingly improved. Figure 8 shows the confusion matrix of four control models at the 12 dB SNR. The figure shows that the basic model RN has more severe confusion for modulation signals 16QAM and 256QAM, as well as modulation signals AM-DSB-SC and AM-DSB-WC. The addition of class reconstruction modules and multi-level comparison operations has somewhat alleviated their confusion. This proves that our design concept is correct and effective, the addition of class reconstruction modules enhances the ability to accurately express potential features of samples, while multi-level comparison operations can make comprehensive decisions on the abstract expression of samples at different levels, thereby improving the decision-classification ability of the model.

### 5.4. Comparison of Recognition Performance under Different C-Way K-Shot Settings

This section conducted experimental exploration of different values of *C* and *K* to further verify the impact of the number of categories *C* extracted during the construction of the meta task and the number of samples *K* extracted for each category on the recognition accuracy of the algorithm proposed in this paper.

**Figure 9:**
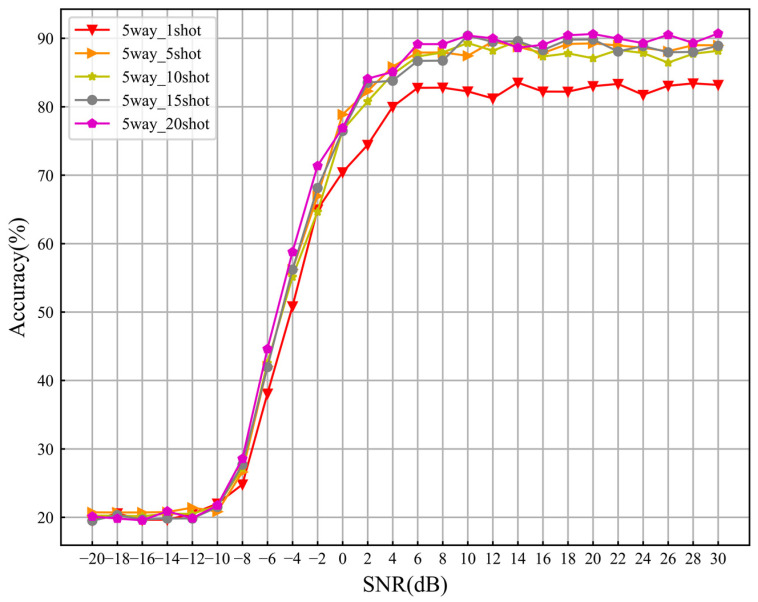
Recognition accuracy curves of models under different *K* values.

Firstly, we fix *C* = 5 and then take *K* = 1, 5, 10, 15 and 20 for experiments. As shown in Figure 9, the recognition performance of the model varies with the SNR under different sample sizes. From the graph, it can be seen that as the *K* value increases, the recognition performance of the model also improves further. According to the aforementioned theory, the method proposed in this article determines its category by comparing the relation scores between query samples and category features in the metric space. Therefore, changing the *K* value for training and testing will definitely impact recognition performance. More specifically, the metric-based theory suggests that each category has a feature embedding in the metric space that is far from each other. The mean of each category’s support sample feature vectors is used in the relation network as the class feature embedding. As the *K* value increases, the estimation of class feature embedding becomes more accurate, resulting in smaller recognition errors when further determining the category of the test sample, thereby improving the overall recognition performance. When the value of *K* increases from 1 to 5, there is a significant improvement in recognition performance. However, when *K* > 5, further increasing the value of K has a more gradual effect on recognition performance. Specifically, when *K* increased from 1 to 5, the average recognition accuracy of the model increased from 61.54% to 65.98% and the performance improved by 4.44%. When *K* increased from 5 to 20, the average recognition accuracy of the model increased from 65.98% to 66.86%, and the recognition performance only improved by 0.88%. This also indicates that the algorithm proposed in this article does not require many supporting samples, but it can still achieve excellent recognition performance.

**Figure 10:**
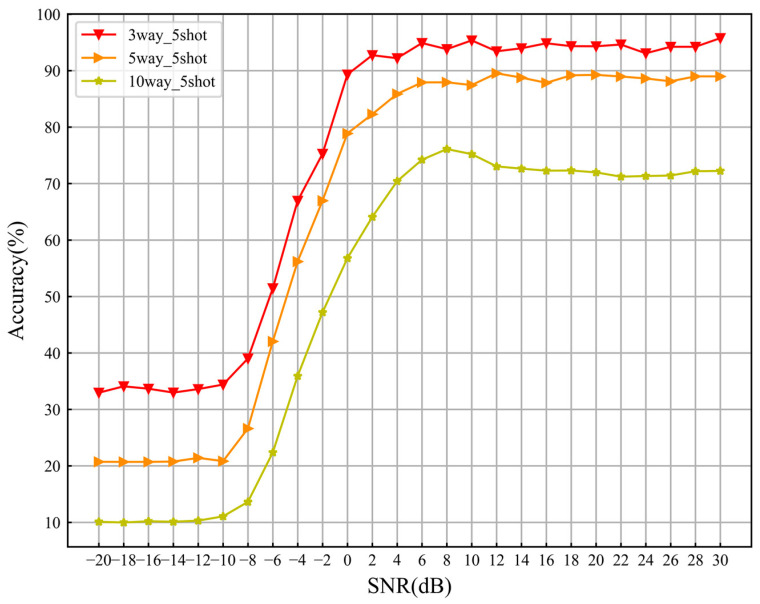
Recognition accuracy curves of models under different *C* values.

Secondly, fix *K* = 5 and conduct experiments with *C* = 3, 5 and 10 to verify the impact of the number of supporting sample categories *C* on recognition performance. Figure 10 shows the model-recognition accuracy variation curve with SNRs for different *C* values. It can be seen from the figure that as the *C* value increases, the overall recognition performance of the model decreases. When *C* increased from 5 to 10, the overall recognition accuracy of the model decreased from 65.98% to 50.70%, and the recognition performance decreased by 15.28%. We believe that as the *C* value increases, i.e., the number of supporting categories increases, the decision space for model category inference will become larger. On the one hand, this increases the difficulty of learning the model. On the other hand, it increases the difficulty of judging the relation between query samples and supporting categories in the metric space, leading to a decrease in the overall recognition performance of the model.

## 6. Conclusions

This article proposes a novel meta-learning method called Multi-level Comparison Relationship Network (MCRN-CR) with class reconstruction to address the few-shot dilemma in AMR. In this method, a multi-level comparison relation network structure is designed, which outputs feature maps through embedding functions, comprehensively calculates the relationship scores between query samples and support samples and determines the modulation category. At the same time, a class reconstruction module is introduced for the embedding function in the network. An autoencoder is used to reconstruct the supporting samples, and its encoder is used as the embedding function. The meta-learning training paradigm is used to train the model, combining classification error and reconstruction error. The experimental results on the RadioML2018 dataset show that MCRN-CR can significantly alleviate the small sample problem in AMR, and its performance is superior to existing methods. This study provides new ideas for solving AMR problems under few-shot conditions and has important application value in related fields such as wireless security. However, it should be acknowledged that in the experiment of this article, the category spaces of the source domain and the target domain are both small. Therefore, in future work, we will collect more diverse training samples from the source domain to support the model’s training and achieve good recognition performance on the target domain test set with a larger category space.

## Figures and Tables

**Table 1:** Structure of relationship module.

Layer	Structure
RM1	Conv1d (out_channels = 64, kernel_size = 4, stride = 2) ReLU + BN
RM2	Conv1d (out_channels = 64, kernel_size = 4, stride = 2) ReLU + BN
RM3	Conv1d (out_channels = 64, kernel_size = 4, stride = 2) ReLU + BN
RM4	Conv1d (out_channels = 64, kernel_size = 4, stride = 2) ReLU + BN
RM5	Conv1d (out_channels = 64, kernel_size = 4, stride = 2) ReLU + BN

**Table 2:** Dataset partitioning.

Dataset Name		Modulation Category	Sample Quantity
Source domain	Training set	128QAM, 32PSK, 16APSK, 32QAM, FM, GMSK, 32APSK, 64QAM, BPSK, 8PSK, AM-SSB-SC, 4ASK, 16PSK, 64APSK	14 × 24 × 1000
Target domain	Support subset	128APSK, AM-DSB-SC, AM-SSB-WC, QPSK, 256QAM, AM-DSB-WC, OOK, 16QAM, OQPSK, 8ASK	10 × 24 × 20
	Test set	128APSK, AM-DSB-SC, AM-SSB-WC, QPSK, 256QAM, AM-DSB-WC, OOK, 16QAM, OQPSK, 8ASK	10 × 24 × 980

**Table 3:** Comparison of average recognition accuracy of different methods.

Model	−20:2:−2 dB	0:2:30 dB	−20:2:30 dB	Highest Accuracy
MCRN-CR	31.69%	87.41%	65.98%	89.25%
AMR-CapsNet [42]	19.22%	44.42%	34.73%	46.40%
AMCRN [40]	32.13%	83.09%	63.49%	86.25%
PN [8]	30.93%	83.00%	62.98%	85.41%
RN [9]	30.72%	84.08%	63.56%	86.68%
AMR-CVNN	20.16%	81.55%	57.94%	85.04%

**Table 4:** Comparison of average recognition accuracy of different control models.

Model	−20:2:−2 dB	0:2:30 dB	−20:2:30 dB	Highest Accuracy
MCRN-CR	31.69%	87.41%	65.98%	89.25%
MCRN	31.42%	86.24%	65.15%	88.15%
RN-CR	31.02%	85.16%	64.34%	87.80%
RN	30.72%	84.08%	63.56%	86.43%

## Data Availability

The data presented in this study are available on request from the corresponding author.
